# T2 values of the acutely infarcted myocardium following primary PCI: the relationship with infarct characteristics and gadolinium extracellular volume of distribution

**DOI:** 10.1186/1532-429X-17-S1-P138

**Published:** 2015-02-03

**Authors:** Elisa McAlindon, Amardeep Ghosh Dastidar, Nauman Ahmed, Chris B Lawton, Tom Johnson, Julian Strange, Andreas Baumbach, Chiara Bucciarelli-Ducci

**Affiliations:** Bristol Heart Institute, Bristol, UK; Heart and Lung Centre, New Cross Hospital, Wolverhampton, UK

## Background

T2 mapping has been shown to detect myocardial oedema as assessed by histology in animals, and is a promising sequence to detect the area at risk in STEMI. The specific cut off value to identify abnormal myocardium in STEMI has not been identified nor have the effects of infarct characteristics on T2 valves. The extra cellular volume fraction (ECV) has been validated against histology in humans. The aims of this study were to identify a cut-off T2 for prediction of myocardial oedema following acute reperfused myocardial infarction and determine if infarct characteristics affect T2 values. In addition, this study aims to determine if T2 values are associated with the ECV following STEMI.

## Methods

50 consecutive patients presenting with STEMI to the Bristol Heart Institute Primary PCI service were approached for inclusion in the study. Patients had a CMR scan day 2 following STEMI. T2 values were assessed by T2 mapping. The myocardium was divided according to the AHA segments. Regional wall motion, LGE transmurality, presence of persistent microvascular obstruction (PMVO) and T2 values were assessed per segment. Segments were deemed affected if they did not have normal wall motion. In addition, a further 30 patients had a CMR day 2 following STEMI. Pre-contrast T1, T2 valves were assessed. Post contrast T1 was measured at 25 minutes following contrast administration. ECV was calculated as: Myocardial ECV = (1−hematocrit) × (ΔR1myocardium/Δ R1blood), where R1=1/T1. All patients gave written informed consent. The study was approved by the local ethics committee.

## Results

**Figure 1:**
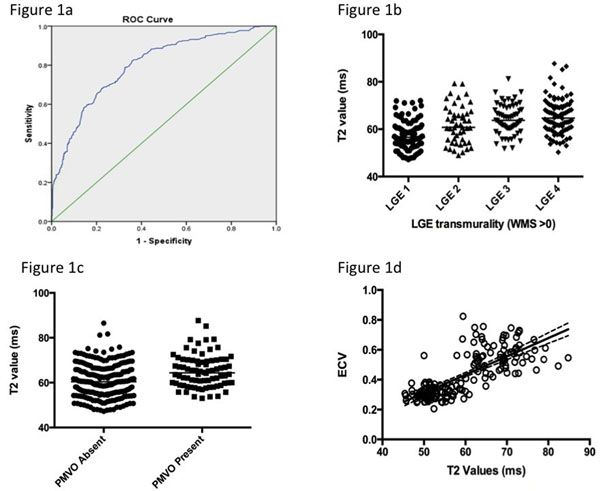
a) ROC T2 values, 1b) T2 values and LGE transmurality, 1c) T2 values and PMVO, 1d) T2 values and ECV.

A T2 value of 55ms most optimally differentiated the segments with regional wall motion abnormalities from those segments with a normal systolic function (remote segments, T2 61 ms), with a sensitivity of 0.79 and a 1-specificity of 0.33 (AUC 0.802, p<0.001)(Figure 1a). T2 values differ depending on the transmurality of the LGE per segment: LGE 1 (0-25%) median 56.6ms, IQR 53.9-60.1ms; LGE 2 (25-50%) median 60.1ms, IQR 55.0-67.0ms; LGE 3 (50-75%) median 63.7ms, IQR 60.3ms-69.4ms; LGE 4 (75-100%) median 64.7 ms, IQR 60.0-69.8ms p<0.0001(Figure 1b). The presence of persistent microvascular obstruction also affected the T2 values: in infarcted myocardium without PMVO (median 60.4 ms, IQR 55.1-65.8ms) T2 values in infarcted myocardium with PMVO (median 64.4ms, IQR 59.7-70.0 ms), p<0.0001(Figure 1c). There was a good association between native T1 values and T2 values (r= 0.785, r^2^=617, p< 0.001). There was a significant association between T2 values and ECV measured at 25 minutes following contrast administration (r=0.76, r^2^= 0.57, p<0.0001)(Figure 1d).

## Conclusions

The optimal cut off value to determine affected myocardium following acute myocardial infarction is 55ms. However, this must be taken in context with the knowledge that infarct characteristics can affect T2 values. There is a significant association between both native T1 and T2 values with a significant association between T2 values and ECV.

## Funding

This study was funded by the National Institute for Health Research Biomedical Research Unit in Cardiovascular Disease at the University Hospitals Bristol NHS Foundation Trust and the University of Bristol.

